# Pet Co-Sleeping and Well-Being: Evidence from Two Cross-Sectional Online Surveys of Youths and Adults

**DOI:** 10.3390/clockssleep8020025

**Published:** 2026-05-07

**Authors:** Kaori Endo, Keiichi Shimatani, Norimichi Suzuki

**Affiliations:** 1 Center for Preventive Medical Sciences, Chiba University, Chiba 263-8522, Japan; 2 Department of Home and Palliative Care Nursing, Graduate School of Health Care Sciences, Tokyo 113-8510, Japan; 3 Design Research Institute, Chiba University, Chiba 131-0044, Japan

**Keywords:** adolescent, adult, cats, co-sleeping, dogs, human-animal interaction, pets, well-being

## Abstract

While the health benefits of pet ownership are well-documented, research on co-sleeping with pets has yielded conflicting results, often contrasting objective sleep disturbances with subjective satisfaction. This study examined the association between dog or cat co-sleeping and well-being across two age groups: adults and youths. Data were collected through two cross-sectional online surveys involving adults (*n* = 2675) and youths (*n* = 1050). Participants reported their pet ownership, co-sleeping status, and well-being using the five-item World Health Organization Well-being Index (WHO-5). Analysis of covariance (ANCOVA) was used to compare WHO-5 scores among three groups: non-owners, owners who do not co-sleep, and co-sleepers. In adults, dog co-sleepers exhibited significantly higher well-being scores compared to non-owners (*p* = 0.025). However, no significant associations were observed in the youth sample. These findings suggest that while pet co-sleeping is often perceived as disruptive, it may be positively associated with subjective well-being in adult populations. Further longitudinal research is needed to clarify the causal relationship and the specific mechanisms underlying this “pet effect” in the context of shared sleep environments.

## 1. Introduction

The benefits of physical and psychological health with pet ownership have long been discussed under the broad notion of the “pet effect.” If the magnitude of these benefits is influenced by the amount of contact time and physical proximity to pets, co-sleeping with pets—defined as sharing either a bed or a bedroom—may represent a particularly important daily context in which close and prolonged human–animal interaction occurs. Although most evidence on pet co-sleeping has been generated in Western countries, existing studies suggest that pet co-sleeping is relatively common, with prevalence varying by country and age group. In one Australian adult sample, at least approximately 10% reported sleeping with their pets [1], while a recent U.S. study reported that 47.6% of adults co-slept with pets [2]. Among children and adolescents, reported prevalence has ranged widely across studies, including 28.4% in Austria [3], 34.6% in Canada [4], 41.7% in the US [5], and 78% in Australia [6]. Does co-sleeping with pets have health benefits?

Prior research on pet co-sleeping has focused primarily on sleep-related outcomes. As summarized in a scoping review, the literature has suggested a recurring pattern in which subjective sleep assessments tend to be more favorable, whereas objective sleep measures are more likely to indicate sleep disruption or poorer sleep [7]. In adults, actigraphy-based studies have shown temporal coupling between dog movement and human movement during sleep [8,9]. In children and adolescents, objective findings generally suggest that pet co-sleeping may be associated with some aspects of less favorable sleep (e.g., longer sleep onset latency or shorter sleep duration/efficiency) [4,5,6]. Importantly, subjective sleep experiences do not always align with objective sleep physiology. For example, in a study using both accelerometry and sleep diaries, dog movement was associated with owner movement, yet the dog-related sleep disruption was not always consciously memorized by the owner [9]. In contrast, questionnaire- and interview-based studies have also reported that people who sleep with pets often describe feelings of comfort, relaxation, and security [10]. As studies using scales of satisfaction and interference from sleeping with pets have shown, people who sleep with pets may derive benefits such as security and safety through pathways different from those measured by the Pittsburgh Sleep Quality Index (PSQI) [11]. As noted in previous research [6], the presence of a pet during the vulnerable process of sleep may serve to dissipate psychological arousal, thereby promoting greater emotional stability. These findings raise the possibility that pet co-sleeping may confer psychosocial benefits through pathways not fully captured by conventional sleep metrics alone.

Well-being is a broader health-related construct than sleep and may therefore provide an important complementary outcome for understanding the overall implications of pet co-sleeping. Given that pets are often regarded as family members and that co-sleeping may be linked to comfort and emotional security, examining the association between pet co-sleeping and well-being is warranted. In addition, well-being has been reported to show a U-shaped pattern across the life course [12], suggesting the value of examining this association across a broader age range rather than within a single developmental stage. Furthermore, prior studies suggest that associations between pet ownership and well-being may differ by animal species (e.g., dogs vs. cats) [13,14], indicating the need to avoid treating “pets” as a homogeneous category.

This study specifically focuses on the broad psychological benefits of human–animal interaction, aiming to elucidate how the presence of pets in the sleep environment relates to overall subjective well-being. In this study, we used data from two online cross-sectional surveys conducted in Japan (a youth survey and an adult survey) to examine the association between dog/cat co-sleeping and well-being. We aimed to provide foundational evidence on a health outcome beyond sleep in relation to pet co-sleeping and to inform public health communication for households living with pets.

## 2. Results

### 2.1. Adult Survey

As shown in Table 1, Of 2675 adult participants, approximately half were male (54.3%). The average age was 45.7 (standard deviation [SD] = 10.7). The prevalence of dog owners was 9.9% and cat owners was 9.5%. Of dog owners, 44.5% reported they slept with their dog. Of cat owners, 44.1% reported they slept with their cats.

**Figure 1:**
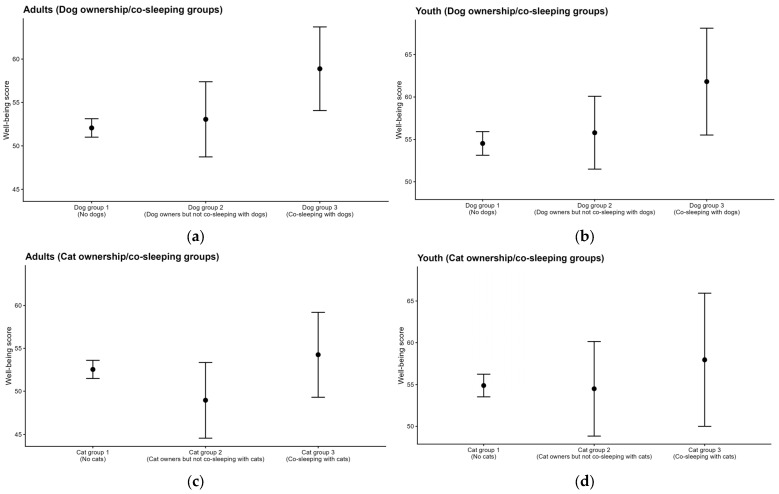
Well-being across dog and cat ownership/co-sleeping groups in adults and youths. Points represent estimated marginal means (EMMs) adjusted for age and sex (and household income in the adult survey), and error bars indicate 95% confidence intervals. (**a**) Adults (Dog ownership/co-sleeping groups), (**b**) Youths (Dog ownership/co-sleeping groups), (**c**) Adults (Cat ownership/co-sleeping groups), and (**d**) Youths (Cat ownership/co-sleeping groups).

As shown in Figure 1, Using the adult survey data (complete-case *n* = 2675), we conducted an ANCOVA with well-being as the dependent variable, dog ownership/co-sleeping status as a fixed factor, and age, sex, and income as covariates. Dog ownership/co-sleeping status was significantly associated with well-being after adjustment for these covariates, F (2, 2669) = 3.69, *p* = 0.025. In Bonferroni-adjusted post hoc comparisons based on estimated marginal means, the co-sleeping dog-owner group (adjusted mean = 58.88) reported higher well-being than the non-dog owner group (adjusted mean = 52.08; mean difference = 6.80, *p* = 0.021), whereas the other pairwise contrasts were not statistically significant. We also conducted an ANCOVA with well-being as the dependent variable, cat ownership/co-sleeping status as a fixed factor, and age, sex, and income as covariates. Cat ownership/co-sleeping status was not significantly associated with well-being after adjustment, F (2, 2669) = 1.48, *p* = 0.227, and no Bonferroni-adjusted pairwise differences between cat ownership/co-sleeping groups reached statistical significance.

### 2.2. Youth Survey

Of 1050 youth participants, exactly half were male. The average age was 16.8 (SD = 0.9). The prevalence of dog owners was 13.4% and cat owners was 8.0%. Of dog owners, 31.9% reported they slept with their dog. Of cat owners, 33.3% reported they slept with their cats.

Using the youth survey data (complete-case *n* = 1050), we conducted an ANCOVA with well-being as the dependent variable, dog ownership/co-sleeping status as a fixed factor, and age and sex as covariates. Dog ownership/co-sleeping status was not significantly associated with well-being after adjustment for age and sex, F (2, 1045) = 2.54, *p* = 0.080. In Bonferroni-adjusted post hoc comparisons based on estimated marginal means, none of the pairwise differences reached statistical significance; however, the co-sleeping dog-owner group (adjusted mean = 61.81) tended to report higher well-being than the non-dog group (adjusted mean = 54.53; mean difference = 7.28, *p* = 0.081). We also conducted an ANCOVA with well-being as the dependent variable, cat ownership/co-sleeping status as a fixed factor, and age and sex as covariates. Cat ownership/co-sleeping status was not significantly associated with well-being after adjustment, F (2, 1045) = 0.29, *p* = 0.748, and no Bonferroni-adjusted pairwise differences between cat ownership/co-sleeping groups were statistically significant.

## 3. Discussion

This study examined the association between pet co-sleeping and well-being across adults and youths, considering the animal species dogs and cats. Approximately 1 in 10 participants owned a dog; 9.9% in the adult survey and 13.4% in the youth survey. The prevalence of cat ownership was also similar, 9.5% in the adult survey and 8.0% in the youth survey. More than 40% of adult pet owners co-slept with their pets (dog co-sleeping 44.5% and cat co-sleeping 44.1%). Among youth pet owners, more than 30% of them co-slept with their pets (dog co-sleeping 31.9% and cat co-sleeping 33.3%). Across two age groups, dog co-sleeping showed a consistent direction of association with well-being—significant in adults and suggestive in youths—while cat co-sleeping was not associated with well-being.

The prevalence of pet ownership in this study among adults (9.9% for dogs and 9.5% for cats) is near the report from another nationwide adult survey in Japan (8.5% for dogs and 8.4% for cats) (National survey on the management of pet dogs and cats 2025 by Japan Pet Food Association). The pet co-sleeping prevalence among adults reported in previous research was mostly from Western countries, e.g., Australia [1] and the US [2], ranging from about 10 to 50%. Also, the prevalence among youths was largely from Western countries, e.g., Austria [3], Australia [6], Canada [4], and the US [5], and ranged from about 30% to 80%. This study added new knowledge on pet co-sleeping prevalence from non-Western countries: about 40% among adult owners and 30% among youth owners in Japan.

Dog co-sleeping was shown to be associated with high well-being among adult owners in this study; however, prior studies on pet co-sleeping have reported mixed sleep-related findings. Research often showed more favorable subjective sleep but more disrupted objective sleep. The existing literature has revealed negative effects of pet co-sleeping on objective sleep conditions, including more movements caused by pets, disturbed sleep onset, interrupted sleep duration, etc. [4,5,6,8,9]. These are understandable considering the mismatch of sleep patterns between humans’ monophasic sleep and dogs’/cats’ polyphasic sleep. Crepuscular habits of dogs and cats may also affect humans’ sleep by inducing early-morning awakening. While these are objective disadvantages on sleep, previous research also reported secure feelings and contentment among pet co-sleepers [10,11]. Such a discrepancy between objective sleep metrics and subjective well-being is consistent with evidence suggesting that physiological sleep disruption does not always align with subjective satisfaction [8,11]. Furthermore, the observed associations may be influenced by the specific Japanese context, where sleeping arrangements such as futons on the floor might moderate the physical transmission of pet movements differently than the mattress-based bed-sharing common in Western studies. According to the differences between dogs and cats, one of the previous studies found poorer sleep in dog co-sleeping than cat co-sleeping [2]. In the other study, conversely, dog co-sleepers felt comfort and security, but cat co-sleepers did not [11]. While research results remain inconclusive, our finding that dog co-sleeping was linked to higher well-being in adults may reflect species-specific patterns of human–animal interaction that confer emotional security. One such pattern may involve the behavioral and physiological synchrony between dogs and their owners. For instance, a previous study in Europe reported a significant correlation between the chronotypes of dogs and humans, suggesting that dogs tend to synchronize their daily activity rhythms with those of their owners [15]. Such rhythmic alignment could potentially minimize nocturnal disruptions and reinforce the emotional bond, providing a plausible explanation for why dog co-sleeping was specifically associated with enhanced well-being in this study. Furthermore, it is plausible that dog co-sleepers may engage in more frequent or consistent dog walking. This increased physical activity and greater exposure to daylight—both of which are independently associated with improved mood and sleep regulation—might further contribute to the enhanced well-being observed in this group. The weaker evidence in youths may be attributable to limited statistical power due to smaller co-sleeping groups (45 dog co-sleepers in the youth survey vs. 118 dog co-sleepers in the adult survey) and to contextual constraints (e.g., parental control on youths’ sleep settings) that shape sleeping arrangements differently from adults’.

To interpret the result in this study, which is high well-being among dog co-sleepers, we should keep in mind that the existing literature reports negative impacts on our sleep. From a sleep-quality perspective, sleep disruption has been repeatedly reported in prior research and should be acknowledged. From a well-being perspective, co-sleeping with dogs may have benefits. Co-sleeping with pets may enhance well-being by increasing opportunities for close physical proximity and affiliative contact at bedtime. In fact, a recent study revealed that touching and being touched by pets has a positive effect on subjective well-being [16]. From the viewpoint of a shared environment between a pet and the owner, owners who sleep with their pets may be more likely to avoid unfavorable (bed)room conditions for their pets. External factors such as light, sound, and temperature are important for sleep. Maintaining these factors properly may lead to a comfortable living setting. Finally, it should be noted that the cross-sectional nature of this study precludes definitive conclusions regarding the direction of causality. While we emphasize the potential benefits of co-sleeping on well-being, the reverse may also be true; individuals with higher baseline well-being and psychological leeway may be more predisposed to choose co-sleeping with their pets. This possibility suggests that co-sleeping might be a ‘proxy’ for a more stable and supportive living environment that inherently fosters well-being. Therefore, such bidirectional relationships between co-sleeping behavior and psychological states warrant careful consideration. Specifically, a large-scale study in Japan has shown that dog owners are significantly more likely to live in owned houses and have higher household incomes [17]. Such stable housing and financial resources may not only independently enhance subjective well-being but also provide the physical environment and psychological security necessary to practice co-sleeping. Such a bidirectional relationship should be further explored in future research.

This study has notable strengths, including the use of two age-diverse surveys, a balanced gender ratio, and a three-level ownership/co-sleeping classification analyzed separately for dogs and cats. However, several limitations should be considered. First, this study is cross-sectional and thus cannot examine any causal effects. Second, this study is not population-based, thus making it hard to generalize the results. Third, the covariates are limited to age and sex only (income is added to the adult survey though). This highlights a need for more comprehensive socio-demographic data and psychological profiles. For instance, personality traits (e.g., extroversion), attachment styles, and more specific socioeconomic factors such as housing stability—as touched upon in our discussion—could significantly influence both co-sleeping choices and self-reported well-being. Inclusion of these factors, as seen in recent studies in the U.S., which have incorporated factors such as race, ethnicity, education level, and marital status [2,5]. The number of people in households—such as whether an individual lives alone or not—could be an important factor. Fourth, co-sleeping with the same bed or room was not provided. Fifth, people sleep with not only a bed but also a futon in Japan; however, this is not examined in this study. Sixth, no information for the number or size of pets was obtained. Seventh, this study lacks objective measures of sleep quality (e.g., actigraphy). As noted in our discussion, subjective well-being and objective sleep parameters do not always align. Future research could integrate both wearable-based objective tracking and subjective psychological scales to more clearly disentangle whether the ‘pet effect’ on well-being is mediated by physiological sleep quality or by perceived emotional security. Future population-based longitudinal studies with richer covariate adjustment and more detailed sleep arrangement measures (e.g., same bed vs. same room, bedding type, number/size of pets) are needed to clarify temporal directionality and potential mediating mechanisms.

## 4. Materials and Methods

### 4.1. Participants and Procedure

Participants were recruited for a cross-sectional observational study of health and well-being. Study inclusion criteria were fluency in Japanese and being ≥18 years old (for the adult survey) or high school students (for the youth survey). There were no exclusion criteria. The study protocol was approved by the Research Ethics Committee of the Graduate School of Medicine, Chiba University (approval number: M10381 for adult survey and M10931 for adolescent survey). We recruited participants through online survey recruitment platforms (Cross Marketing Inc. (Tokyo, Japan) for the adult survey and LINE Research by LY Corporation (Tokyo, Japan) for the youth survey). After providing informed consent online, participants completed a self-report questionnaire assessing their pet ownership, well-being, and demographic characteristics. Participants with pets also reported their sleeping arrangement with their pets. Participants received financial compensation for completing this study that depended on the platform used to access the survey. Data were collected from July to September 2025 for the adult survey and January 2026 for the youth survey.

### 4.2. Materials

#### 4.2.1. Co-Sleeping with Pets

Participants were asked about their dog/cat ownership (yes/no). Participants were asked whether they shared a bed or bedroom with their pets. In line with this study’s focus on overall proximity, these responses were analyzed collectively as ‘co-sleeping’ without further distinguishing between bed-sharing and room-sharing. Participants were categorized into three groups: no dogs (dog group 1), dog owners but not co-sleeping with dogs (dog group 2), and co-sleeping with dogs (dog group 3). Also, they were categorized into three groups: no cats (cat group 1), cat owners but not co-sleeping with cats (cat group 2), and co-sleeping with cats (cat group 3).

#### 4.2.2. Well-Being

Participants answered a five-item World Health Organization Well-being Index (WHO5). The items of the WHO-5 are as follows: “I feel cheerful and in good spirits”; “I feel calm and relaxed”; “I feel active and vigorous”; “I wake up feeling fresh and rested”; and “My daily life is filled with things that interest me.” Each item assessed the degree of well-being over the past two weeks on a six-point Likert-type scale ranging from 0 (at no time) to 5 (all of the time). Total scores derived from the WHO5 ranged from 0 to 25, with higher scores indicating better well-being. The total raw score ranging from 0 to 25 was multiplied by 4 to obtain the final score, with 0 representing the worst imaginable well-being and 100 representing the best imaginable well-being.

#### 4.2.3. Demographic Characteristics

Participants reported their age (continuous) and sex (categorical: male and female). Adult participants also reported their annual household income (ordinal: less than 4 million JPY, 4 million to 10 million JPY, more than 10 million JPY).

### 4.3. Data Analysis

A one-way between-subjects analysis of covariance (ANCOVA) was performed to examine the difference in well-being across the no dog/cat group, dog/cat owner but not co-sleeping group, and dog/cat co-sleeper group, controlling for age and sex (with the addition of income for the adult survey). Post hoc comparisons were conducted using Bonferroni correction. All analyses were performed using the R software (version 4.5.1; R Foundation for Statistical Computing, Vienna, Austria). *p*-values <0.05 were considered statistically significant.

## 5. Conclusions

In conclusion, dog co-sleeping was associated with higher well-being in Japanese adults, while no clear association was observed for cat co-sleeping, and evidence in youths was suggestive but inconclusive. These findings motivate longitudinal research to determine when and for whom pet co-sleeping supports well-being without compromising sleep health.

## Figures and Tables

**Table 1:** Demographic characteristics of participants of the adult survey and youth survey.

		Adult Survey		Youth Survey	
		(*n* = 2675, 100%)		(*n* = 1050, 100%)	
		*n*	%	*n*	%
Dog ownership/co-sleeping condition					
Group 1	(no dogs)	2410	90.1	909	86.6
Group 2	(dog owners but not co-sleeping with dogs)	147	5.5	96	9.1
Group 3	(co-sleeping with dogs)	118	4.4	45	4.3
Cat ownership/co-sleeping condition					
Group 1	(no cats)	2421	90.5	966	92
Group 2	(cat owners but not co-sleeping with cats)	142	5.3	56	5.3
Group 3	(co-sleeping with cats)	112	4.2	28	2.7
Well-being score		Mean = 52.4	SD = 26.9	Mean = 55.0	SD = 21.6
Age		Mean = 45.7	SD = 10.7	Mean = 16.9	SD = 0.9
Sex					
	Male	1452	54.3	525	50
	Female	1223	45.7	525	50
Annual household income (JPY)					
	<4 million	913	34.1		
	4–<10 million	1365	51		
	≥10 million	397	14.8		

## Data Availability

The authors do not have permission to share data.

## Abbreviations

The following abbreviations are used in this manuscript:

WHO-5	the five-item World Health Organization Well-being Index
ANCOVA	analysis of covariance
